# Quorum Sensing and Virulence of *Pseudomonas aeruginosa* during Lung Infection of Cystic Fibrosis Patients

**DOI:** 10.1371/journal.pone.0010115

**Published:** 2010-04-12

**Authors:** Thomas Bjarnsholt, Peter Østrup Jensen, Tim Holm Jakobsen, Richard Phipps, Anne Kirstine Nielsen, Morten Theil Rybtke, Tim Tolker-Nielsen, Michael Givskov, Niels Høiby, Oana Ciofu, the Scandinavian Cystic Fibrosis Study Consortium

**Affiliations:** 1 Institute for International Health, Immunology and Microbiology, University of Copenhagen, Copenhagen, Denmark; 2 Department of Clinical Microbiology, University Hospital, Rigshospitalet, Copenhagen, Denmark; 3 BioSys, Technical University of Denmark, Lyngby, Denmark; Abramson Research Center, United States of America

## Abstract

*Pseudomonas aeruginosa* is the predominant microorganism in chronic lung infection of cystic fibrosis patients. The chronic lung infection is preceded by intermittent colonization. When the chronic infection becomes established, it is well accepted that the isolated strains differ phenotypically from the intermittent strains. Dominating changes are the switch to mucoidity (alginate overproduction) and loss of epigenetic regulation of virulence such as the Quorum Sensing (QS). To elucidate the dynamics of *P. aeruginosa* QS systems during long term infection of the CF lung, we have investigated 238 isolates obtained from 152 CF patients at different stages of infection ranging from intermittent to late chronic. Isolates were characterized with regard to QS signal molecules, alginate, rhamnolipid and elastase production and mutant frequency. The genetic basis for change in QS regulation were investigated and identified by sequence analysis of *lasR*, *rhlR, lasI* and *rhlI*. The first QS system to be lost was the one encoded by *las* system 12 years (median value) after the onset of the lung infection with subsequent loss of the *rhl* encoded system after 17 years (median value) shown as deficiencies in production of the 3-oxo-C12-HSL and C4-HSL QS signal molecules respectively. The concomitant development of QS malfunction significantly correlated with the reduced production of rhamnolipids and elastase and with the occurrence of mutations in the regulatory genes *lasR* and *rhlR.* Accumulation of mutations in both *lasR* and *rhlR* correlated with development of hypermutability. Interestingly, a higher number of mucoid isolates were found to produce C4-HSL signal molecules and rhamnolipids compared to the non-mucoid isolates. As seen from the present data, we can conclude that *P. aeruginosa* and particularly the mucoid strains do not lose the QS regulation or the ability to produce rhamnolipids until the late stage of the chronic infection.

## Introduction

The onset of the chronic lung infection with *Pseudomonas aeruginosa* in CF patients is preceded by intermittent colonization [1] usually with environmental strains [2]. The chain of events leading to the establishment of a persistent infection is mainly due to the biofilm forming capacity of *P. aeruginosa* with important contributions from individual virulence factors such as elastase [3], LPS [4], rhamnolipids [5] and alginate [6]. We have demonstrated that rhamnolipid plays a major role in the defense against the cellular components of the immune system, especially against the polymorphonuclear neutrophilic leukocytes (PMNs) which dominate the immune response in the CF lung [7]–[9]. *P. aeruginosa* respond to the presence of PMNs by upregulating synthesis of a number of virulence determinants including rhamnolipids, all of which are able to cripple and eliminate cells of the host defense which support a ‘launch a shield’ model by which rhamnolipids surround the biofilm bacteria and on contact eliminate incoming PMNs [9].

Production of several *P. aeruginosa* virulence factors is coordinated by a cell density monitoring mechanism termed Quorum Sensing (QS) [10]–[12]. *P. aeruginosa* employ two dominating QS system the *las* and the *rhl* encoded system. Both systems feature specific signal molecules for separation of the processes, 3-oxo-C12-HSL and C4-HSL respectively. The basic AHL QS system is comprised of an I gene encoding the AHL synthetase and a R gene encoding the receptor. During the growth of the bacteria, system specific signal molecules are produced by the synthetase, the I protein. The signal molecules produced by the bacteria bind to the receptor, the R-protein, the AHL-responsive transcriptional activator. The regulator proteins contain two functional domains. The signal molecule binding region, which is located in the N-terminal portion of the protein and a helix-turn-helix motif (HTH) located in the C-terminal, which is responsible for the protein binding to the target promoters [13]–[15]. Within these systems a third analogous receptor, the QscR operates with 3-oxo-C(12)-HSL to modulate gene expression of a specific regulon which overlaps with the two other *las* and *rhl* regulons [16]. *P. aeruginosa* has an additional QS regulatory pathway termed the Pseudomonas quinolone signal (PQS) system [17]. *In vitro* the QS systems of *P. aeruginosa* have been shown to be hierarchically arranged, with the *las* system on top, controlling the *rhl* system [18] and the PQS system positioned as a mediator functionally positioned between the *las* and *rhl* systems. However, it has been proposed that the *rhl* system can be activated independently of the *las* system, and it has been suggested that PQS system controls this activation [17]. This was further substantiated in a recent paper, where the authors provided evidence that *rhl* system is able to overcome the absence of the *las* system by activating specific LasR-controlled functions, including production of 3-oxo-C(12)-HSL and PQS [19].

When the chronic lung infection in CF patients is established it is well recognized that *P. aeruginosa* isolated from the sputum differ phenotypically from the initial intermittent strains even though they produce similar pulse field gel electrophoresis patterns and therefore are considered isogenic [20], [21]. Loss of epigenetic regulatory systems such as QS is one of the dominating changes that occur during the adaptive process of the bacteria in the CF lung [22]. Different models accounting for the selection of non-functional QS systems have been reported. One such model focuses on the special nutrient availability in the CF lung which *P. aeruginosa* has to adapt to [22]. This model is supported by comparison of the genomes obtained from different CF isolates [23] suggesting that *P. aeruginosa* has the potential to act in a range of environmental conditions. Furthermore, the authors suggest that the bacterium acquires or discards genomic segments in order to optimize its genomic repertoire for the present specific environment. Another model focuses on the fact that *P. aeruginosa* is exposed to oxygen radicals which in turn induce genetic mutations [23], [24]. We have recently demonstrated that the polymorphonuclear leukocytes (PMNs) are the major contributors of oxygen radicals in CF sputum [25]. It is therefore likely that oxygen radicals are derived from the PMNs. Recently, the cooperative behavior of mixed populations of bacteria has been studied using populations including both QS wild-type and *lasR* mutants [26]. These studies have introduced the concept of “cheaters” (the QS mutants) exploiting the functional QS systems of other members of the population [26], [27]. It might be that in CF lungs, although *P. aeruginosa lasR-* mutants may accumulate, QS-active members of the population are still maintained for the benefit of all members of the bacterial community.

Based on these observations we aimed to correlate the changes that occur in the QS systems with expression of virulence during stages of intermittent and chronic lung infections in CF patients. The capability to produce 3-oxo-C12-HSL and C4-HSL signal molecules and the sequences of *lasR* and *rhlR* encoding the receptor-transcriptional regulators as well as the *lasI* and *rhlI* encoding the synthethases were investigated in a large number of randomly collected CF isolates, (pairs of mucoid and non-mucoid if available) obtained from the intermittent or chronic stages of lung infection. The dynamics of the functionality of QS systems in the clinical strains were correlated to rhamnolipids and elastase production as well as to the mutational frequencies of the isolates. Our results show that functionality of the *rhl* encoded system is maintained longer than the *las* system during the chronic infection, especially in the mucoid isolates, providing evidence for the possible role of QS inhibitors in the treatment of early as well as late stages of *P. aeruginosa* infections.

## Results and Discussion

### QS functionality and duration of infection

Loss of QS regulation is generally considered a hallmark of chronic virulence and has been described for several *P. aeruginosa* CF isolates [22], [28]. To investigate the dynamics of the QS loss at different stages of the *P. aeruginosa* lung infection, we determined the production of QS signal molecules of isolates collected from patients with intermittent colonization as well as chronic lung infection at different time points.

**Figure 1:**
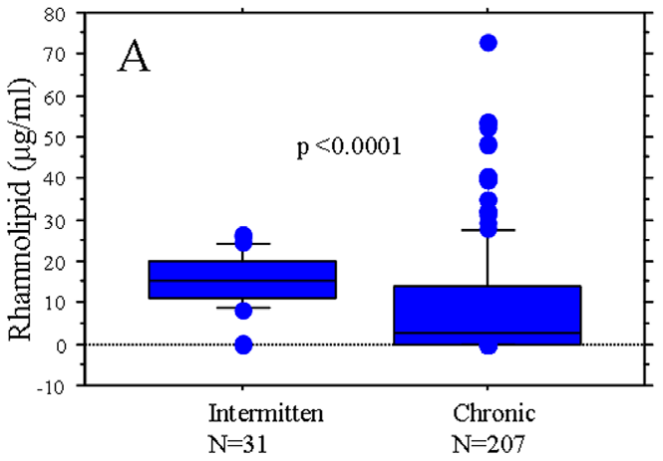
Distribution of *in vitro* rhamnolipid production in *P. aeruginosa* isolates from CF patients at different stages of lung infection. The box and whisker plots represent the median (thick line inside the box), 10, 25, 75 and 90 centiles of the *in vitro* rhamnolipid production (µg/ml) of *P. aeruginosa* isolates from intermittently colonized and chronically infected patients; Mann-Whitney test was used to investigate the significance of the difference between the groups.

The *P. aeruginosa* CF isolates from the intermittently colonized patients showed significantly higher frequency of strains with simultaneous production of both QS molecules (χ^2^ test p<0.0001) and higher levels of rhamnolipid production (median [ranges] = 15.3[0–26.4] µg/ml) compared to the isolates from the group of chronically infected patients (median [ranges] = 2.4[0–72.8] µg/ml)(Mann-Whitney, p<0.0001) (figure 1).

The CF isolates were divided in four groups according to their ability to produce QS molecules: a group producing both 3-oxo-C12-HSL and C4-HSL (n = 58), a group producing only C4-HSL (n = 63), a group producing only 3-oxo-C12-HSL (n = 16) and a group not producing QS molecules (n = 73). A significant difference was found between the duration of the chronic lung infection of the CF patients harboring the isolates belonging to the different groups (table 1). The majority of the CF isolates (63) were not producing 3-oxo-C12-HSL after 12 years of infection while only a small proportion (16 isolates) were 3-oxo-C12-HSL producers but lost the ability to produce C4-HSL. Importantly, the lost of both QS molecules was found first after 17 years of infection. This shows that the abilities to produce 3-oxo-C12-HSL and C4-HSL signal molecules are lost at different time points during the chronic lung infection and particularly interesting is the finding of C4-HSL molecules in isolates from the late stages of the infection. This indicates that the *rhl* system is functional even in the late phases of the chronic lung infection and suggests that the Las-independent regulation of *rhl* system is maintained during the chronic lung infection. These data emphasize that the shielding through rhamnolipid production might play an important role during the first 17 years of infection.

**Table 1:** Duration of the chronic lung infection of CF patients harboring *P. aeruginosa* isolates producing both C4-HSL and 3-oxo-C12-HSL, either C4-HSL or 3-oxo-C12-HSL or none of the QS molecules and distribution of the rhamnolipid and elastase levels in CF *P. aeruginosa* isolates producing both, one or none of the QS molecules.

	C4-HSL + 3-oxo-C12-HSL+ (n = 58)	C4-HSL + 3-oxo-C12-HSL - (n = 63)	C4-HSL –3-oxo-C12-HSL + (n = 16)	C4-HSL –3-oxo-C12-HSL - (n = 72)
Duration of chronic infection(years) Median[ranges]	7.5* [intermittent-29]	12** [intermittent-32]	13 [1]–[32]	17* ^,^ ** [intermittent-31]
Rhamnolipid (µg/ml) Median[ranges]	16.6^a^^,^ b ^,^ d [0–53.8]	10.7^a^^,^ c [0–72.8]	1.7^d^ [0–11]	0^b^^,^ c [0–48]
Elastase activity (mU) Median[ranges]	48^1^ [0–276]	40.3 [0–180]	46.7 [0–109]	36.8^1^ [0–104.8]

*p = 0.0002.

**p = 0.016 (Mann-Whitney).

a p = 0.008.

b p<0.0001.

c p<0.0001.

d p<0.0001 (Mann-Whitney).

1 p = 0.01 (Mann-Whitney).

Significant differences in the level of rhamnolipid (table 1) and elastase (table 1) were found between the four groups of QS signal molecule producers concurring that these virulence factors require a functional QS system for expression.

### QS and mucoidity

**Figure 2:**
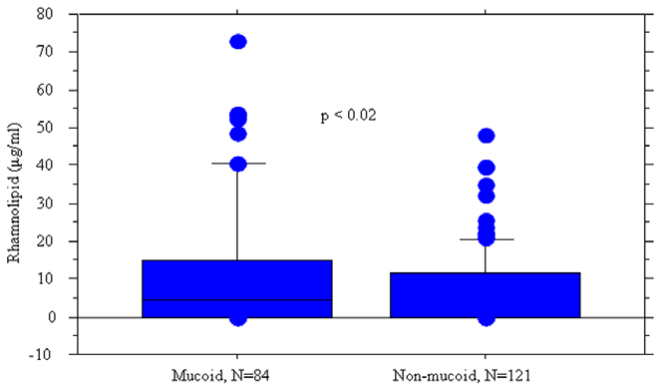
Distribution of rhamnolipid production in mucoid and nonmucoid isolates. Box and whisker plots represent the median (thick line inside the box), 10, 25, 75 and 90 centiles of the *in vitro* rhamnolipid production (µg/ml) of mucoid and non-mucoid *P. aeruginosa* isolates from chronically infected CF patients. Mann-Whitney test was used to investigate the significance of the difference between the groups.

Early occurrence of mucoid *P. aeruginosa* in the sputum of CF patients has been correlated to a poor prognosis [5], [29]. Mucoidity has been shown to be selected for in the CF lung due to the protective role of alginates against oxygen radicals from activated PMNs [30]. In addition, as judged from flow-cell and animal experiments, mucoid isolates form more robust biofilms[31], [32]. Investigations of QS functionality and connected phenotypes expressed by mucoid and non-mucoid isolates that were obtained from the chronically infected patients, showed that a significantly higher proportion of mucoid isolates produced C4-HSL compared to their non-mucoid counterparts (χ^2^ test, p = 0.02). Furthermore, this number was found to correlate with significantly higher amounts of rhamnolipids (median [ranges] = 4.5 [0–72.8] µg/ml) produced by mucoid compared with non-mucoid isolates (median [ranges] = 0[0–48]), (p = 0.02, Mann-Whitney) (Figure 2).

Thus, mucoid isolates may be protected against the antimicrobial properties of the PMNs not only by alginate but also due to the production of rhamnolipids. This is in accordance with recent data from an animal model of chronic lung infection which showed that persistence against the host defense was maintained in mucoid but lost in nonmucoid isolates during the chronic lung infection of one CF patient [33]. This difference in the functionality of the QS system between mucoid and non-mucoid isolates strongly support that different adaptation strategies are employed by the two phenotypes [5], [31].

### QS and clonal distribution

The typing analysis showed that three previously identified bacterial clones entitled DK-1 (39 isolates), DK-2 (29 isolates) and NO (26 isolates) were represented among the 238 CF isolates. Clones DK-1 or “red” and DK-2 or “blue” are two dominant clones in the Copenhagen CF Center and clone NO is a clone identified among the Norwegian isolates[32]–[34]. The rest of the isolates were considered non-clonally related.

**Figure 3:**
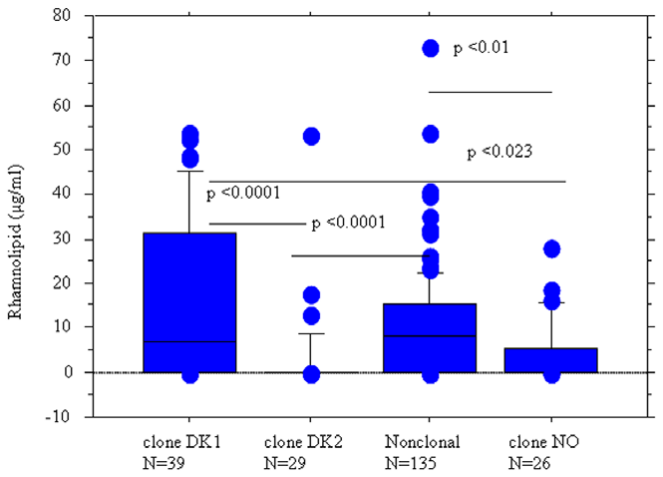
Distribution of rhamnolipid production in isolates belonging to different clones. Box and whisker plots represent the median (thick line inside the box), 10, 25, 75 and 90 centiles of the *in vitro* rhamnolipid production (µg/ml) of *P. aeruginosa* isolates belonging to different clones included in the study. The clones DK1 and DK2 are the two dominating clones in Denmark. Clone NO is a dominating clone in Norway and the non-clonal isolates have no clonal relationship. Mann-Whitney test was used to investigate the significance of the difference between the groups.

Significant differences in the functionality of the QS systems were found among the various clonal groups. While functionality loss of both the *las* and *rhl* systems was found in 75% of the DK-2 strains, this phenotype was encountered in only 46% and 33% of the NO and DK-1 isolates, respectively. These differences in the ability of the isolates to produce QS signal molecules were associated with significant differences in the ability of the isolates to produce rhamnolipids (Figure 3). Importantly, we always saw a positive correlation between production of C4-HSL and rhamnolipids but no correlation between 3-oxo-C12-HSL and rhamnolipid production. This is in accordance with findings by us [35] and others who showed that PAO1 do not require a functional *las* system for expression of *rhl* and *pqs* controlled genes.

Several isolates belonging to the DK-2 clone did not harbor a *lasR* gene as shown by the lack of gene amplification which in turn suggests that this particular mutant of the DK-2 clone might have spread among CF patients after the apparent loss of QS signal recognition, or that a deletion hotspot exists at the particular chromosomal position. However, this second option is statistically very unlikely. Similar results were obtained with isolates belonging to the NO and DK-1 clones, although these isolates were found to harbor a functional *rhl* system. This suggests that dissemination and establishment within a community of CF patients does not require a functional LasR- system. Alternatively, LasR proficient bacterial subpopulations might have been present in the initial infection but these subpopulations were not identified in our study. However, it is important to mention that we identified two CF patients that harbored QS-proficient DK-2 bacteria, suggesting that several evolutionary lineages develop in the CF population as recently published by Wilder [36].

### QS signal molecules and sequence of the QS genes

To investigate the cause of QS loss we performed sequence analysis of the genes *lasR* and *rhlR* encoding the QS-regulators LasR and RhlR as well as of the genes *lasI* and *rhlI* encoding the signal molecule synthetases LasI and RhlI, respectively. The analysis showed that the wild-type sequences of *lasI* were conserved among the CF *P. aeruginosa* isolates. From 238 isolates, we only found a single occurrence of a loss of function mutation in *lasI* gene and intact *lasR* gene. However, the vast majority of the isolates presented point mutations in *rhlI* (data not shown) especially C249A leading to D83E which interestingly has also been found in isolates from CF patients attending the Oregon Health and Science University [36]. The measurements of C4-HSL were not affected by these point mutations. This indicates that these point mutations have no effect on the functionality of the gene and its encoded product. Mutations preferentially occurred in the genes encoding the regulatory proteins, in accordance with previous observations [37].

**Figure 4:**
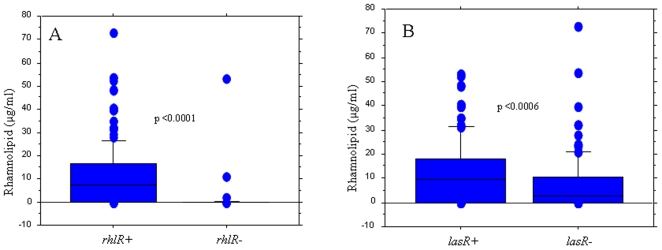
Distribution of rhamnolipid production in isolates with or without mutations in the QS regulatory genes. Box and whisker plots represent the median (thick line inside the box), 10, 25, 75 and 90 centiles of the *in vitro* rhamnolipid production (µg/ml) of *P. aeruginosa* isolates (A) with (*rhlR−*) or without mutations (*rhlR+*) in the regulatory gene *rhlR*; (B) with (*lasR−*) or without mutations (*lasR+*) in the regulatory gene *lasR* Mann-Whitney test was used to investigate the significance of the difference between the groups.

CF isolates with mutations in the regulatory genes *lasR* or *rhlR* produced significantly less 3-oxo-C12-HSL (χ^2^ p<0.0001) and C4-HSL (χ^2^ p<0.0001) respectively and lower levels of rhamnolipids compared to the isolates with wild-type genes (Figure 4A and 4B).

**Figure 5:**
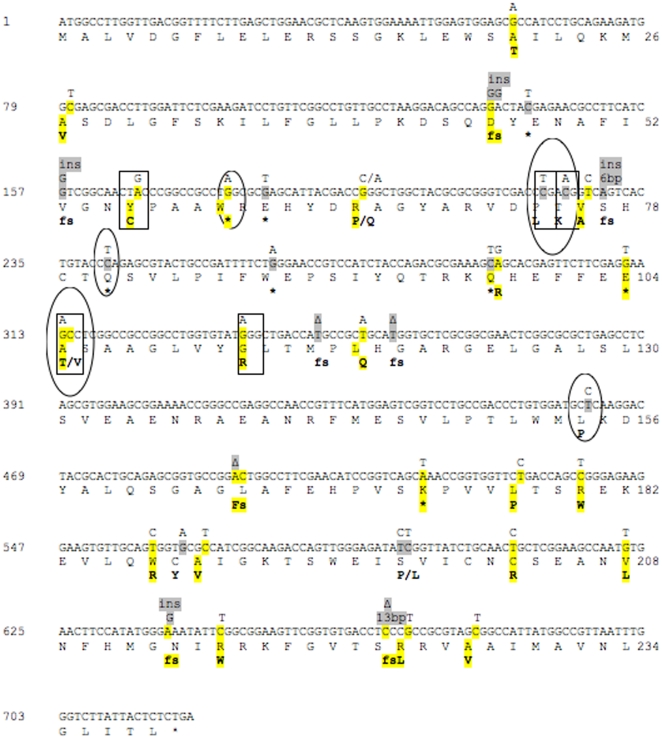
Mutations in the *lasR* gene (A) and *rhlR* gene (B) of *P. aeruginosa* isolates from CF patients. The nucleotide sequence alterations were identified by alignment with the PAO1 sequence. On top of the PAO1 sequence the nucleotide substitutions, insertions (ins) or deletions (Δ) are indicated. Under the amino acid sequence, frame shifts (fs), stop codons(*) or amino acid deletions (Δ) or changes are indicated in bold. The altered nucleotides are shown in yellow if the mutation was complemented with a plasmid containing the wild-type *lasR* or in grey if not complemented. Amino acid changes that have been previously shown to impair the LasR function [37], [38] are marked in squares and amino acid changes that have been previously described in *in vitro* studies [22], [39], [48] are encircled.

**Figure 6:**
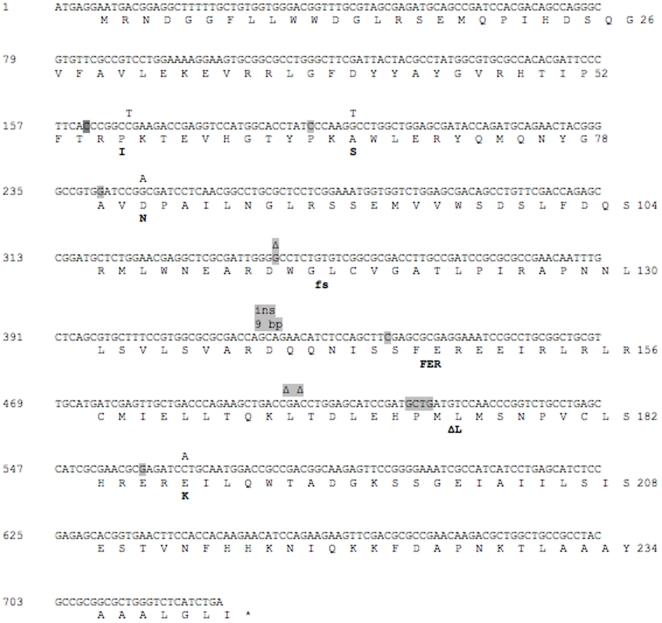
Mutations in the *rhlR* gene of *P. aeruginosa* isolates from CF patients. The nucleotide sequence alterations were identified by alignment with the PAO1 sequence. On top of the PAO1 sequence the nucleotide substitutions, insertions (ins) or deletions (Δ) are indicated. Under the amino acid sequence, frame shifts (fs), stop codons(*) or amino acid deletions (Δ) or changes are indicated in bold.

The type of mutations identified in *lasR* and *rhlR* genes are presented in Figure 5 and 6. Mutations in *lasR* were identified in both the signal-binding N-terminal domain and the DNA-binding domain (C-terminal). The mutations observed (Figure 5) were insertions and deletions leading to frame shifts and point mutations (both transitions and transversions) resulting in either stop codons or substitutions in conserved, semi-conserved, or non-conserved amino acids [38]. The complementation assays showed that the identified point mutations were responsible for the phenotypes (marked in yellow in figure 5). In the signal-binding domain, particular interesting are the mutations causing a Tyr56 to Cys exchange and a Thr75 to Lys exchange as both Tyr56 and Thr75 have been shown to be important for the binding of signal molecules to LasR [39]. In addition, Pro74, Ala105 and Gly113 were all amino acids that have been described as important for the multimerization and function of LasR (marked by squares in figure 5) [38]. Several of the mutations described in this study have been found by other investigators in *lasR* mutants of *P. aeruginosa* obtained under *in vitro* evolution experiments (encircled in figure 5) [22], [27], [38], [40]. This reflects a level of similarity between the *in vitro* and *in vivo* bacterial evolution and suggest a possible selective advantage of these kinds of mutations *in vivo*.

The *lasR* gene could not be amplified in 32 out of 39 DK-2 isolates suggesting the deletion of this gene in this particular clone at the time of investigation. Loss of function mutations in the *rhlR* gene were identified in 24 out of 39 DK-2 isolates. The most frequently encountered mutation was a 3 bp deletion at position 519 (4 isolates) or 520 (18 isolates) with loss of Leu 173. These gene changes explained the basis for the loss of both 3-oxo-C12-HSL and C4-HSL signal molecules in the DK-2 clone.

### Mutations in QS genes and mutability of the isolates

**Figure 7:**
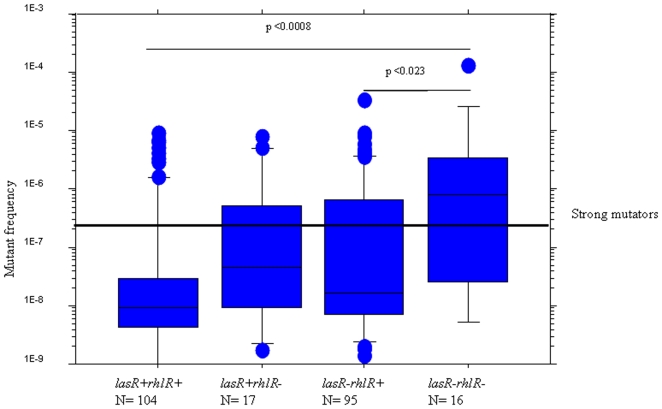
Association between occurrence of mutations in the QS genes and the mutability of the isolates. Box and whisker plots represent the median (thick line inside the box), 10, 25, 75 and 90 centiles of the mutation frequency measured by the occurrence of spontaneous antibiotic resistance in isolates with mutations in both QS regulatory genes (*lasR−rhlR−*), only in one of the regulatory genes (*lasR−rhlR+*) or (*lasR+rhlR−*) or without mutations in either of the regulatory genes (*lasR+rhlR+*). Mann-Whitney test was used to investigate the significance of the differences between the groups.

Increase in mutation frequencies leading to a weak mutator phenotype of the isolates was found to correlate with the loss of functionality of either *lasR* or *rhlR* (Figure 7) and occurred after a mean of 15 years. However, strong mutators occurred late during chronic infection (mean 19.7 years) and correlated to accumulation of mutations in both *lasR* and *rhlR* (Figure 7). These data are in accordance with previous observations showing that *lasR* mutants of *P. aeruginosa* obtained in *in vitro* evolution experiments were not hypermutable [27]. We propose that mutations in either of the QS regulatory genes can occur in isolates with non- or weak mutator phenotype, followed in time by the occurrence of strong mutators with increased accumulation of mutations which disables the entire QS system. This sequence of events in the CF lung might be explained by an impaired protection of the QS deficient strains against the mutagenic effects of reactive-oxygen species liberated by activated PMNs due to their decreased production of catalase and superoxide-dismutase.

### Conclusion

The current knowledge regarding the chronic *P. aeruginosa* infection of the CF lung suggests that *P. aeruginosa* adjust its phenotypes to the environment of the CF lung. We have previously proposed that QS and especially the production of rhamnolipids are important for the initial stage of infection providing the bacteria with an immune shield which is protective against the antimicrobial activity of PMNs. As seen from the present data we can conclude that *P. aeruginosa* isolates may lose LasR dependent QS but keep the capability of RhlR dependent QS regulation enabling production of a number of important host damaging virulence factors particularly rhamnolipids. This capability is maintained till late in the chronic infection, in particular in mucoid isolates. Our results show that the mucoid isolates are protected from the antimicrobial activity of PMNs in the CF lung by both alginate which act as a ROS scavenger but also by their ability to produce rhamnolipids which provide a shield against cellular components of the innate immune response. This also suggest that a treatment with drugs interfering with QS and in particular the lower hierarchy of QS regulated virulence factors such as rhamnolipids might be useful not only in the early stages of the infection but also in the treatment of chronic infections with QS producing strains.

## Materials and Methods

### CF patients and bacterial strains

In the present study, 238 *P. aeruginosa* isolates obtained from 152 Scandinavian CF patients have been investigated. The random collection consist of 31 non-mucoid isolates from intermittently colonized patients, 35 non-mucoid isolates from chronically infected patients and 86 pairs of mucoid and non-mucoid isolates (172 isolates) from chronically infected patients. The 152 patients were distributed in five CF centers in Scandinavia as follows: Copenhagen, 22 intermittently and 62 chronically infected patients; Aarhus, 13 chronically infected patients; Lund, 9 intermittently and 16 chronically infected patients; Uppsala, 11 chronically infected patients and Oslo, 19 chronically infected patients. The mean duration of the lung infection in chronically infected patients was 15 years (from 1 and up to 32 years).

The CF patients were considered chronically infected when *P. aeruginosa* was cultured in the sputum for six consecutive months or serum precipitating antibodies to *P. aeruginosa* ≥2 by crossed-immuno-electrophoresis [41].

Sputum samples obtained by expectoration or endolaryngeal suction were Gram-stained and examined under the microscope to confirm the origin from the lower airways with the exception of the samples from Norway. The sputum samples of the 152 Scandinavian CF patients were plated on Blue agar plates (a modified Conrad Drigalski medium selective for Gram-negative rods, Statens Serum Institute, Copenhagen, Denmark containing peptone 10 g, yeast extract 5 g, NaCl 5 g, agar 11 g, detergent 0,05 g, Sodium thiosulphate 1 g, bromthymolblue 0,1 g, lactose 9 g and glucose 0,4 g).

When mucoid and non-mucoid *P. aeruginosa* isolates were simultaneously isolated from sputum samples, both phenotypes were collected from the Blue agar plate (Gram negative selective growth media).

### Genotyping by pulsed-field gel electroforesis (PFGE)

All isolates were typed by PFGE as described previously using *SpeI* enzyme [42], [43]. After PFGE, the band patterns were visualized by ethidium bromide staining and then photographed (GelDoc™ imaging system, Bio-Rad, Munich). The patterns were analyzed by Fingerprinting™ II software, Bio-Rad, CA, USA). The clonal relatedness of the individual pairs of mucoid and non-mucoid *P. aeruginosa* was confirmed according to Tenover [44]. Isolates with PFGE patterns that differ from each other by two to three bands were considered clonally related, as this pattern is consistent with a single genetic event, *i.e*. a point mutation or an insertion or deletion of the DNA. Isolates with PFGE patterns that differed by more than three bands were considered to belong to different strains.

### Measurement of mutant frequencies in cultures of *P. aeruginosa* isolates

Mutation frequencies can be determined via fluctuation analysis [45]. However, because fluctuation analyses are laborious and the present study comprised 238 *P. aeruginosa* isolates, we took advantage of the fact that mutation frequencies and mutant frequencies are proportional in sufficiently large cultures. To determine mutant frequencies each bacterial isolate was grown overnight in 100 ml Luria-Bertani (LB) medium, upon which 20 ml culture was centrifuged at 3,000 rpm for 10 min, and resuspended in 1 ml LB medium. A 100 µl volume of undiluted, 10^−1^ diluted and 10^−2^ diluted was plated on LB plates containing 300 µg/ml rifampicin and on LB plates containing 500 µg/ml streptomycin. A 100-µl volume of 10^−7^ to 10^−10^ dilutions was plated on LB plates. After incubation at 37°C for 48 h the numbers of CFU were counted, and the frequencies of rifampicin resistant and streptomycin resistant mutants were calculated.

According to the mutant frequencies the isolates were grouped in strong mutators (20 times the mutant frequency of PAO1 (≥2×10^−7^), weak mutators (>1×10^−8^<2×10^−7^) or nonmutators [46], [47].

### DNA sequence analysis of *lasR, rhlR, lasI* and *rhlI* genes

*lasR, rhlR, lasI* and *rhlI* genes from all isolates were PCR amplified using the primer sets described below. After purification (Promega Wisart purification kit, Madison,USA) the PCR products were sequenced on a Macrogen automatic DNA sequencer ABI3700. The number of reads was between two and four for each gene of each strain. The sequencing results were compared with the strain PAO1 sequence (www.pseudomonas.com) with DNASIS Max vesion 2.0 (Hitachi software Engineering), in order to determine the occurrence of sequence variations.

Complementation of the *lasR* mutations in the clinical *P. aeruginosa* isolates was done by electroporation of plasmid MH645 (Plac-lasR cloned in BamHI site of pBBR1-MCS5 [48]). The success of complementation was verified by the reestablishment of the protease activity.

For PCR amplification and sequencing the following primers were used.

*lasR* start 5′- ATGGCCTTGGTTGACGGTT-3′

*lasR* stop 5′-GCAAGATCAGAGAGTAATAAGACCCA-3′

*lasI* start 5′- ATGATCGTACAAATTGGTCGGC-3′

*lasI* stop 5′- GTCATGAAACCGCCAGTCG-3′

*rhlR* Fw 5′GCCATGATTTTGCCGTATCGG-3′

*rhlR* rev 5′- CGAGCATGCGGCAGGAGAAGC-3′

*rhlI* Fw 5′- GGAGTATCAGGGTAGGGATGC-3′

*rhlI* rev 5′- CGAGCATGCGGCAGGAGAAGC-3

### Determination of elastase activity, rhamnolipid and signal molecule production

Bacteria from −80°C freeze stocks were plated onto blue agar plates (State Serum Institute, Denmark) and incubated at 37°C overnight. From each plate (representing an isolate) one colony was selected and grown as a overnight culture in either ox-broth (for elastase activity) or AB trace minimal medium containing 3 mM glucose (32) at 37°C with shaking (rhamnolipid and signal molecules production). The supernatant was either dialyzed against sterile water (for elastase activity) or sterile filtered using 0.2 µm pore filters (16543; Sartorius), frozen and kept for further analysis.

#### Elastase activity

Elastase activity was determined in a spectrophotometric assay using elastin-Congo red (Sigma) as a substrate, as previously described [49].

#### Rhamnolipid production

Liquid chromatography/electron spray ionization mass spectrometry (LC-ESI-MS) data on pure rhamnolipid was used to produce a standard curve for rhamnolipid B (concentration vs. total ionization current (TIC)). The rhamnolipid standards used for calculating the concentration curve were analyzed immediately prior to, as well as after analysis of the samples, in order to minimize potential differences in ionization levels of rhamnolipid between the samples. Rhamnolipid concentrations were normalized to the standard curve for rhamnolipid B. In the analysis the total rhamnolipid concentration was derived from the six major rhamnolipids, with the following masses [M+NH4]+: 668,4; 694,4; 696,4; 522,4; 548,4 and 550.4. These equate to C10-C10-rha-rha, an unidentified C10-C12Δ-rha-rha, C10-C12-rha-rha, and the respective mono-rhamnose derivatives. BHPLC-MS analysis was performed with an agilent 1100 series high performance liquid chromatography (HPLC) connected to a micromass LCT TOF MS.

#### Measurement of signal molecules

The measurements for QS signal molecules were performed in “black 96 welled microtiter plates” (Nunc, black PolyBase, USA), using specific QS reporter strains. The reporter strains used were previously described: MH205 (C4-HSL) [50] and MH155 (3oxo-C12-HSL) [51]. ABT minimal media supplemented with thiamin (25 µg/ml), 0.5% glucose and 0.5% casaminoacids was used to grow the reporter strains. For controls 10 mM *N*-butanoyl-L-homoserine lactone (BHL), 10 mM *N-*Dodecanoyl-DL-homoserine lactone (DDHL) and supernatant from a wild type *P. aeruginosa* was used. Overnight cultures of each strain were prepared in 5 ml LB and 5 ml ABT supplemented with 0.5% glucose and 0.5% casaminoacids, respectively. For QS signal molecule measurements, 2 fold dilutions of the sterile filtered cultures were performed with a final volume of 150 µl. To each well 150 µl of the appropriate monitor strain diluted to OD_450_ 0.1, was added.

The plate was then incubated and read in a Multi Label reader (wallac 1320Viktor, Perkin Elmer). Measurements of turbidity at OD_450_ and fluorescence (excitation 485 nm and emission 535 nm) were done every 15 minutes for 17 hours. The temperature was kept at 37°C.

### Ethics

The Danish, Norwegian and Swedish Research Ethics Committees approved the collection of bacteria and informed written consent was obtained from all patients.

### Statistical analysis

The description and analysis of the data were carried out using StatView® 5.0.1. software.

The distribution of the data did not follow in all groups a Normal distribution and therefore the data are presented as median [ranges] and the graphic representation was done by box-plots indicating the median, 10, 25, 75 and 90 centiles of the groups. The dots outside the whiskers represent single isolates with values smaller than in 10% or higher than in 90% of the isolates.

The nonparametric Mann-Whitney test was used for comparison between the different groups. Categorical data were analyzed in frequency tables that fulfilled the guidelines for “ a large sample” approximation and χ^2^ test was used to test the null hypothesis (e.g. QS producers in mucoid vs nonmucoid isolates). The level of significance was 5%.
